# Relative Age Effects and Place of Early Development Constrain Male Youth Italian Swimmers’ Developmental Experiences

**DOI:** 10.3390/sports12110309

**Published:** 2024-11-14

**Authors:** Gabriele Morganti, Adam Leigh Kelly, Matteo Vitarelli, Francesca Strassoldo di Villanova, Bruno Ruscello, Francesca Campoli, Elvira Padua

**Affiliations:** 1 Department of Human Sciences and Promotion of the Quality of Life, San Raffaele Roma Open University, 00166 Rome, Italy; gabriele.morganti@uniroma5.it (G.M.); matteo.vitarelli@uniroma5.it (M.V.); francesca.strassoldo@uniroma5.it (F.S.d.V.); bruno.ruscello@uniroma5.it (B.R.); francesca.campoli@uniroma5.it (F.C.); 2 Research for Athlete and Youth Sport Development (RAYSD) Lab, Centre for Life and Sport Sciences (CLaSS), Faculty of Health, Education and Life Sciences, Birmingham City University, Birmingham B15 3TN, UK; adam.kelly@bcu.ac.uk; 3 Department of Neurosciences, Biomedicine and Movement, University of Verona, 37134 Verona, Italy; 4 Department of Industrial Engineering, Faculty of Engineering, “Tor Vergata” University, 00133 Rome, Italy; 5 LUISS SportLab, LUISS University, 00197 Rome, Italy; 6 Department of Sports Engineering Lab, “Tor Vergata” University, 00133 Rome, Italy

**Keywords:** birth advantages, youth swimming, talent identification, talent development, relative age effects, place of early development

## Abstract

Italian swimming emphasizes the early specialization of selected children from approximately 6 to 7 years old. Such an approach often leads to selection biases (i.e., birth advantages), which may undermine swimmers’ development and progression through the talent pathway. Accordingly, this study aimed to: (a) explore the presence of birth advantages at the annual Italian national age-group competition by observing the birth quarter (BQ) and place of early development (PED) distribution of 514 U15 swimmers; and (b) investigate how birth advantages affect swimmers’ ability to maintain their national status by comparing the BQ and PED distributions of 555 U17 national-level swimmers to the expected values (i.e., U15 distribution). Chi-square statistics for the U15 revealed an overrepresentation of BQ1s and swimmers developing in north and central Italy (*p*-values < 0.0001). In contrast, the U17’s BQ (*p* < 0.001) and PED (*p* = 0.01) distributions appeared skewed compared to the U15, favoring swimmers born in BQ3 and BQ4, and swimmers developing in north Italy (odds ratios: 1.69, 1.76, 1.39 respectively). The findings highlighted that cultural–contextual features of the environment shape Italian youth swimmers’ development and their progression through the current talent pathway.

## 1. Introduction

Italian swimming follows a deterministic model of talent pathway [1], which is founded on a pyramidical system that progresses from prioritizing participation and inclusion at the very first developmental stages (i.e., 3–7 years of age) to an increasing evaluation of children’s level of ability and performance standards. The top of this pyramid emphasizes pre-determined stages of progression, which engage fewer and fewer people [2]. In line with this, the Italian swimming federation (Federazione Italiana Nuoto: FIN) promotes practices of early identification and specialization, whereby ‘talented’ children enrolled in organized swimming activities are identified every year by their respective swimming clubs, and those selected are offered to specialize from approximately 6–7 years of age and onwards. Similar to other sports, the identification of talented youth swimmers generally highlights swimmers displaying ideal body proportions, outperforming their peers, and showing an early ability level [2,3,4], as well as swimmers with the required traits to fit in with the given specialization pathway for the sport in question, such as a love for swimming and competition and a willingness to follow coaches’ instructions [5,6,7].

Past research has investigated talent identification procedures in soccer and suggested that coaches and selectors have their own ideas of what being a competitive player entails, based on which they form an overall understanding of what attributes need to be evaluated [3]. Indeed, selection decisions depend highly on coaches’ preferences and past experiences, with coaches relying on their ‘gut feeling’ [4,8]. Swimming coaches often adopt a similar approach [9] and follow the framework presented by Lund and Soderstrom [4] in soccer, whereby coaches’ selection decisions are guided by their previous identification experiences, as well as by evaluating swimmers’ attitudes and analyzing their early results. As such, coaches (a) understand swimmers’ eventual development by comparing their attributes and qualities to past athletes they recognized as talented, who eventually developed into elite senior swimmers; (b) rank swimmers based on their current performance values (i.e., best times and skill level) through the organization of annual racing contests that pit enrolled children in competition against each other; and (c) assess swimmers’ attitudes by measuring swimmers’ genuine interest in swimming practices and competition.

A recent study from Alexiou et al. [9] explored swimming coaches’ perceptions related to talent identification and development practices. They revealed that their decision-making was highly reliant on their experiential knowledge, which may be, in part, due to a lack of objective and commonly accepted testing procedures for use in youth swimming. In more detail, coaches reported using their ‘coach’s eye’ (i.e., an intuitive, subjective, experience-based, and holistic process of talent selection; see Lath et al. [10] for a discussion on this), to (a) understand swimmers’ holistic (eventual) development; (b) analyze swimmers’ performance level at different developmental ages to predict their (eventual) performance; and (c) assess their desire to train and achieve success to evaluate their trainability and adaptability (i.e., future potential). Moreover, coaches considered the age range of 9 to 12 years to be the ideal age for recognizing talented swimmers, thus reinforcing their perceptions of the need for early identification and specialization practices. This is likely caused, at least in part, by contextual needs (i.e., the lack of pool space), whereby coaches are somewhat required to select talented swimmers early, as they cannot afford to have everyone in their club.

Selection procedures appear to be conducted on the concept of having talent, which Aggerholm [11] described as being linked to an idea of talent that is something individuals have, that is present a priori (i.e., a property; an inner quality related to the ability to outperform one’s peers). In line with this, Alexiou and colleagues [9] reported that most swimming coaches believe a talented swimmer is ‘born’ (rather than developed). A recent review investigating the genetic characteristics of competitive swimmers has revealed several genetic variants that may be linked to swimming expertise, as well as to specialization in short-distance or long-distance swimming events, respectively [12]. However, despite this, the authors also acknowledged the importance of considering how favorable genetic predispositions interact with other environmental and contextual features (i.e., the presence of infrastructures, relationships with coaches and peers, and parental support). Indeed, as presented by Morganti et al. [1], the expression “to have talent” does not describe the existence of any innate characteristics but rather presents the relationship between swimmers’ past experiences (i.e., hours of training, developmental opportunities, coaching and mentoring) and personal characteristics (i.e., relative age, biological maturation, intrinsic motivation), combined with their current performance values and skill levels. In line with this, research has shown that when sports systems select athletes that are able to satisfy early high-performance outcomes, they contribute to the proliferation of several selection biases [13], such as birth advantages.

Birth advantages (i.e., relative age effects (RAEs) and place of early development (PED)) are related to variables present at early ages. These include organizational structures (i.e., grouping children based on their date of birth; [14]), composition effects (i.e., socioeconomic status and characteristics of the inhabitants; [15]), and the community’s physical aspects (i.e., presence of and proximity to infrastructures, access to facilities, presence of high-performance clubs; [13,16]), which have both a short-term influence on athletes’ developmental opportunities and experiences and a long-lasting impact on their future career (and participation) outcomes [17]. Swimming has already been shown to be vulnerable to RAEs [18]. Past studies have highlighted that relatively older swimmers (i.e., those born near the start of the selection cut-off date) have competitive advantages over their peers and that they are indeed overrepresented across the first developmental stages (i.e., 12–14 years [19,20,21,22]) compared to their relatively younger peers (i.e., those born towards the end of the selection cut-off date). However, the research reported inconsistent results related to RAEs’ influence on swimmers’ transition through the talent pathway. For instance, transient effects (i.e., decrements in magnitude at older ages) of relative age have been found in Australian [19] and Portuguese [20,23] cohorts, while no RAEs decrements were recorded in German [21,22] swimmers. This indicates the need to further investigate RAEs’ incidence and their impacts related to specific cultures, as different organizational structures, competition formats, and contextual features interact differently with athletes’ personal characteristics and developmental settings [24].

Past research has shown the geospatial diversity in athletes’ developmental opportunities over several sports, nations, and continents [13,24,25,26,27,28]. Studies have presented associations between a community’s socioeconomic status and physical aspects and children’s early sport-specific experiences, early skill development, and future career outcomes [28,29,30]. For instance, in Italy, Morganti et al. [26,27] revealed how regional disparities interact with and constrain soccer players’ opportunities to develop and their developmental outcomes. Specifically, the authors reported on the Italian ‘southern question’ (i.e., north–south dualism), which is related to multiple socioeconomic and cultural factors, and negatively influences southern Italian players’ likelihood of accessing the greatest developmental environments and completing the youth-to-senior transition. Therefore, further exploration and studies in the area are required to understand the disparities in opportunities to develop and promote equitable talent pathways for everyone, independent of his or her birthdate, birthplace, and/or place of early development. However, no studies have investigated the geographic distribution of swimmers and the presence of contextual constraints in swimming.

Accordingly, this study aimed to examine how birth advantages influence swimmers’ entrance into (Part 1) and progression through (Part 2) the Italian swimming talent pathway. Part 1 focused on investigating the presence of birth advantages in Italian swimmers and their influence on youth swimmers’ likelihood of competing at the annual Under 15 (U15) national age-group competition. Part 2 analyzed how birth advantages interacted with national-level swimmers’ progression through the talent pathway by recording the birthdates and place of early development of Italian youth swimmers competing at the annual U17 national age-group event and comparing them to the expected distribution taken from the U15 cohort. We hypothesized that (a) the highly competitive environment and the highly selective competition formats promoted by the FIN, reflected in the early specialization practices and early success promotion, respectively, lead to the presence of RAEs, whereby early-born swimmers are overrepresented; and (b) the regional disparities that characterize Italy define swimmers’ developmental experiences and their skill-acquisition processes, whereby those developing in north and central Italy are overrepresented.

## 2. Materials and Methods

### 2.1. Subjects

To be eligible for inclusion in this study, a swimmer must have competed in the annual U15 and/or U17 male Italian national swimming competitions during the 2015–2016, 2016–2017, and/or 2017–2018 seasons. The research sample comprised a total of 1067 Italian male swimmers (U15, *n* = 514; U17, *n* = 555), born between 1999 and 2003 (both years included), who were included in the statistical analyses. Since all data were publicly accessible on the internet, no approval by an ethical committee was required.

### 2.2. Procedures

**Figure 1:**
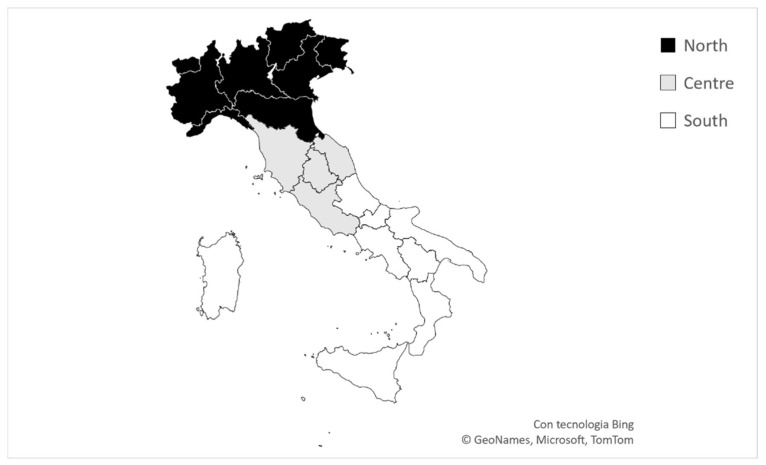
Italian map showing the microregions of Italy divided into macroregions (north, center, and south).

The data for this study (i.e., swimmers’ appearances at the annual age-group national championship, birthdates, and place of early development) were publicly available in the public domain and retrieved online from the FIN website (https://www.federnuoto.it/home/nuoto/graduatorie.html) (accessed on 1 March 2024). Italy’s 20 micro-regions were divided into three macro-regions, as per national indications and policies (i.e., north, center, and south; Figure 1) [31,32], as previously carried out in other studies conducted in the Italian context [26,27]. Swimmers were classified based on birthdate using birth quartiles (i.e., Birth Quarter 1 (BQ1) = January, February, and March; BQ2 = April, May, and June; BQ3 = July, August, and September; BQ4 = October, November, and December) and PED (i.e., northern, central, and southern Italy). The observed birthdate and place of early development distribution of the U15 and the U17 cohorts were calculated and compared to the expected distribution obtained from census statistics [31,32], as well as to the U15 observed distribution to analyze the continuation in the talent model, respectively. 

### 2.3. Data Analysis

In Part 1 of this study, the observed BQ and PED distributions of the U15 cohort were compared to the general population norms distribution obtained from the census data [31,32]. In Part 2, to investigate the progression of youth swimmers through the talent pathway, the BQ and PED distributions were compared to their respective expected distributions, taken from the U15 cohort. A chi-square goodness-of-fit test (χ2) was employed to compare the observed and expected distributions. As chi-square statistics cannot reveal the magnitude and the direction of a relationship when the results are significant, effect sizes (Cramer’s V) and odds ratios (ORs) were also computed. The Cramer’s V was interpreted as follows: values between 0.05 and 0.09 indicated a weak effect size, 0.10–0.14 indicated a moderate effect size, 0.15–0.24 indicated a strong effect size, and 0.25 or more indicated a very strong effect size [33]. The ORs and 95% CIs were used to separately compare the BQs and PEDs of those who entered the annual age-group national championship at the U15 and U17 levels, respectively. ORs were calculated using the youngest group as the reference (BQ4) and included comparisons across macroregions (i.e., north vs. center; north vs. south; center vs. south). CIs that included 1 (i.e., 95% CI 0.90–1.10) were marked as showing no association. The results were considered statistically significant at *p* < 0.05. Statistical analyses were performed, and maps of Italy were created using Microsoft Excel (2019).

## 3. Results

**Figure 2:**
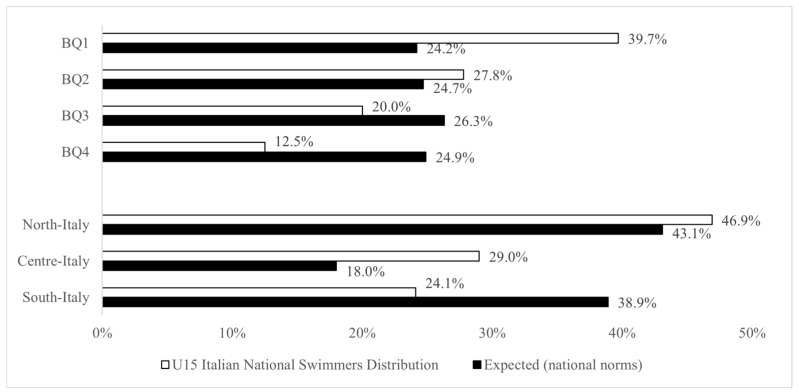
The observed BQ and PED distributions for the cohort of U15 Italian national swimmers compared to the national norms (expected values).

Figure 2 separately displays the observed BQ and PED distributions for the U15 Italian male national-level swimmers, as well as the general population norms.

**Figure 3:**
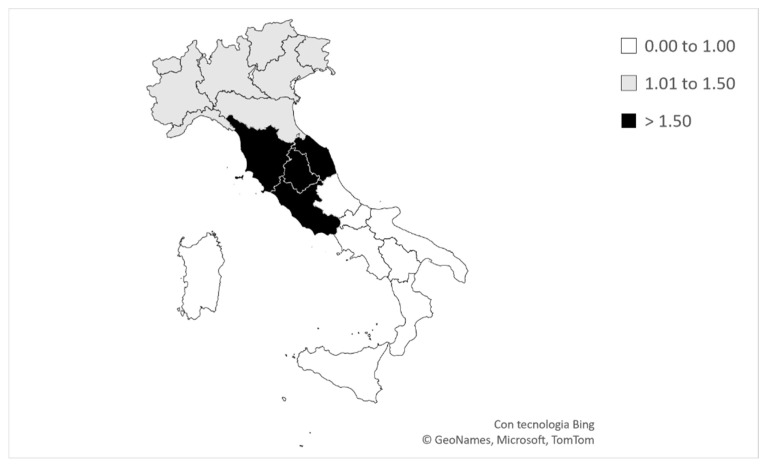
Map of Italy, with microregions separated by lines and colored according to the ORs for competing at the annual U15 Italian male national swimming championship.

The results demonstrated that both the birthdate and place of early development distributions at the U15 level appeared significantly skewed in comparison to their respective general population norms (birthdate: χ2 (3) = 92.7; *p* < 0.001; very strong effect size; place of early development: χ2 (2) = 65; *p* < 0.001; very strong effect size). In this context, the ORs indicated that swimmers born in the first three quarters (BQ1, BQ2, and BQ3) had the highest probability of gaining access to the annual U15 Italian male national competition compared to BQ4 swimmers (ORs BQ1 vs. BQ4 (95% CI) = 3.29 (2.26–4.78); BQ2 vs. BQ4 = 2.25 (1.53–3.30); and BQ3 vs. BQ4 = 1.53 (1.03–2.26)). Similarly, the results showed that swimmers developing in north and central Italy had an increased likelihood of competing in the annual U15 Italian male national competition compared to their counterparts developing in south Italy (ORs north vs. south (95% CI) = 1.75 (1.31–2.34); and center vs. south = 2.60 (1.84–3.66); Figure 3).

**Figure 4:**
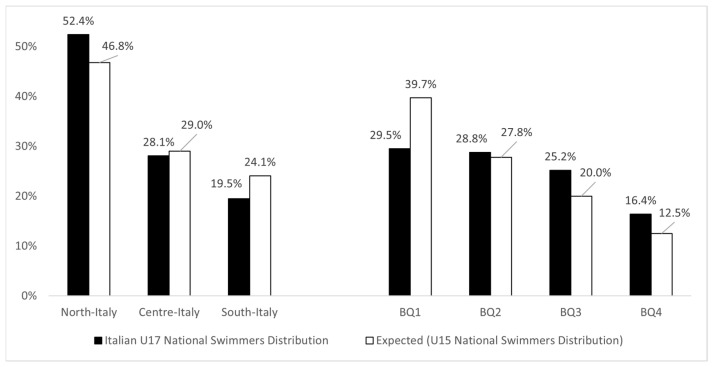
The observed BQs and place of early development distributions for the U17 Italian national swimmers’ cohort in comparison to the U15 distribution (expected values).

Figure 4 separately displays the observed BQ and PED distributions of the U17 cohort, as well as the expected distribution (taken from the U15 cohort). Chi-square statistics indicated that the birthdate and place of early development distributions at the U17 level appeared significantly skewed compared to the U15 cohort, which favored relatively younger swimmers and swimmers developing in northern Italy. Indeed, although older swimmers were still overrepresented, their numbers were lower than expected. Indeed, there was a higher presence of BQ4s compared to the U15 cohort (birthdate: χ2 (3) = 28; *p* < 0.001; moderate effect size; place of early development: χ2 (2) = 8.73; *p* < 0.01; weak effect size).

In line with this, additional statistical analyses underlined that swimmers born in BQ4 and BQ3 recorded the highest likelihood of progressing to next national stage compared to BQ1 swimmers (ORs BQ4 vs. BQ1 (95% CI) = 1.76 (1.12–2.56); and BQ3 vs. BQ1 = 1.69 (1.23–2.34)); whereas swimmers developing in northern regions showed the greatest likelihood of progressing through their youth career (ORs north vs. south (95% CI): 1.39 (1.02–1.88).

## 4. Discussion

Italian swimming promotes early identification and specialization procedures, following a deterministic model of talent development [2]. The research has shown that these practices often lead to the proliferation of several selection biases, such as relative age effects and birthplace effects [34]. Accordingly, this study aimed to explore whether birth advantages influence Italian swimmers’ entrance into and progression through the talent pathway. To the authors’ knowledge, this was the first study to investigate these biases in the Italian swimming context and explore a possible association between birth advantages and progression to the next developmental stage. The findings from Part 1 of this study reported that early-born swimmers and swimmers developing in north and central Italy are overrepresented in the U15 national Italian swimmers cohort. Additionally, Part 2 showed that the U17 national Italian swimmer population appears to be significantly different from the U15 distribution, presenting with more relatively younger swimmers than expected and even more swimmers developing in north Italy. Overall, these findings show how progression through the existing talent model is highly associated with swimmers’ birthdate and place of early development.

Part 1 of this study reported that swimmers’ access to the U15 national under-age competition is dependent on their birthdate and place of early development. The overrepresentation of relatively older swimmers is in line with previous research conducted in the area, which determined the prevalence of RAEs at youth levels for national-level swimmers in German [21,22], Australian [19], and Portuguese [20,23] cohorts. This is due to youth swimming organizations’ tendency to place children as young as 8 years in competition against each other. For instance, the FIN allocates children to regional and national rankings based on their annual best times, which determine their opportunities to race in regional and national annual events. Accordingly, only the fastest swimmers in the respective age groups gain access to the best developmental opportunities and experiences. These competition formats reinforce notions of confrontation and competition among children, coaches, and parents alike, shifting the focus from swimmers’ eventual performance (long-term development) to their early successes and results, and thus leading to the proliferation of early specialization practices (children are induced to specialize in a single or a few swimming strokes) from very early stages. As such, by organizing annual regional and national events in which access is restricted to a few select swimmers able to reach the performance standards imposed upon them in advance by the governing body, the FIN is indirectly favoring the overrepresentation of early-born swimmers, who are more likely to outperform their later-born peers due to the relative advantages of their age [35]. Indeed, they experience initial benefits, such as having more time to practice, compete, and improve, which can lead to the faster and earlier development of sport-specific skills, supporting an early increase in performance outcomes [17].

In this study, following the indications outlined by Hernandez-Simal et al. [29], when analyzing the birthplace effects in our cohorts of swimmers, we decided to include the place of early development as a dependent variable as it is considered more reliable by the latest research conducted in this area [15,36]. The place of early development is described as the environment where the developing athlete develops their relationships and engages in various activities [37]. The results revealed that swimmers developing in north and central Italy recorded the highest likelihood of competing at the national U15 level. Previous research conducted on Italian soccer obtained similar results [26,27]. Therefore, it is suggested that this phenomenon reflects the broader regional contrasts in Italy. Specifically, south Italy is characterized by a lack of sporting infrastructure and a lower socio-economic and cultural status [38]. Indeed, similarly to Italian soccer, access to organized swimming activities in Italy follows the ‘pay-to-play’ model, requiring parental financial support, whereby children’s participation in swimming clubs is tied to their families’ sociocultural and economic status. This could negatively affect children developing in south Italy’s involvement in organized swimming activities. Moreover, the heterogeneous distribution of sporting and swimming infrastructures, reflected in the lack of such facilities in the southern regions [39], requires parents from southern Italy who want to enroll their children in swimming clubs to expend further resources in terms of time and money for transportation [40]. In line with this, the FIN counts nine national training centers: five in the central regions, three in the north, and only one in the south.

Findings from Part 2 of this study recorded a higher presence of BQ4s in the U17 cohort than expected, thus showing that later-born swimmers may be more likely to progress through the talent pathway. Research conducted on swimming has already found transient effects of relative age, highlighting a decrease in its magnitude as swimmers get older (e.g., [19,20,23]), and an inverse effect as swimmers approach the senior stage, reflecting an overrepresentation of relatively younger swimmers [19]. As such, the results obtained from this study (a) provide a further test of the presence of knock-on effects of relative age, whereby RAEs at successive developmental stages can only be attributed to a residual bias [41]; and (b) present a new exploration of the ‘underdog hypothesis’ [42] in the swimming context. Indeed, past studies across different sports and contexts have presented how BQ4s have the greatest likelihood to progress through the developmental pathway [19], complete the youth-to-senior transition [41,43,44,45], and achieve the most career achievements [41,42]. Despite this tendency, the mechanisms that account for these changes remain speculative, although researchers have proposed that multiple factors are involved [19]. In the context of our study, the highly selective competition formats promoted by the FIN influence the way coaches are evaluated (i.e., their ability to produce winning age-group teams [46]), which indirectly affects the environment where youth swimmers are trained (i.e., selection procedures, coaches’ behaviors, and training regiments). Youth swimmers are exposed to more intensive training regiments (i.e., train like ‘mini-adults’, daily sessions, and dry-land sessions) and, following a Darwinian ‘survival of the fittest’ form of development and competition (e.g., [47,48]), are required to conform and comply with conventional standards, which prioritize the ‘right attitude’, and mental disposition (i.e., willingness to train hard and compete; [49,50]). Children unable to meet these expectations and fulfil the requirements set by the governing bodies and their swimming clubs [51] are left out of competitions and receive fewer opportunities to develop (i.e., value-directedness towards working hard). In line with this, Lang [52], in her brilliant ethnographic work conducted on various youth swimming clubs across the United Kingdom, noticed how coaches’ main concern was that athletes were able to comply with their demands (i.e., strict training regiments) and to follow instructions. Therefore, in the long term, the results from our study suggest that the value-directedness towards working hard emphasized by the FIN favors later-born swimmers that are able to continue swimming competitively. Indeed, the higher proportion of BQ4s found in the U17 cohort may be caused by the fact that, through facing the challenges associated with overcoming any age-related differences and having the opportunity to compete against their older peers and fulfill their potential, they acquired a more robust coping mechanism [53], improved their motivation, and developed the required character [17] to comply to organizational demands. Moreover, it is also hypothesized that, to overcome such challenges, BQ4s developed higher technical competencies (e.g., [19,42,54,55]), suggesting that when age-related differences decrease and/or dissipate, they are favored due to their higher skill levels and technical proficiencies [19,56]. However, it is also worth recognizing here that despite the higher presence of BQ4s at the U17 national level than expected from the U15 cohort, later-born swimmers continued to be the least represented. That suggests that the mechanisms of the ‘underdog hypothesis’ likely only benefit a minority that are already in the system, as the presence of later-born swimmers continues to be below national expectations. This indicates that most BQ4s may experience early drop-out. Accordingly, more research is needed to substantiate the relative advantages and effects of age.

The results from Part 2 of this study also revealed a performance gap between swimmers developing in the north compared to their peers in the south when progressing through the next developmental stages (i.e., from U15 to U17). Past research has indicated that the social dimension of talent is an important factor to consider when investigating athletes’ transition. Receiving the required economic [15], affective [38], and institutional [6] support is fundamental for achieving a normative transition (i.e., progression through the model or reaching the senior level [57]). In line with this, when athletes encounter socioeconomic, cultural, political, geographical, and historical barriers, they may experience a non-normative transition [29]. When athletes reach a certain level of performance, they often need to leave their former and local club to join a high-performance club where they can receive the highest developmental support (i.e., access to better training facilities, competent technical staff, expert coaches, and the highest levels of competition) [38,47]. However, the vast majority of swimmers developing in the southern regions, due to the heterogeneous distribution of swimming facilities and high-performance youth swimming clubs, experience a lack of institutional support and may be forced to leave their families or invest further money in transportation to access greater developmental opportunities. As such, the higher prevalence of swimmers developing in the north may be due to talented swimmers’ migration to the numerous high-performance youth clubs there. This aligns with previous research that has highlighted geographical mobility as a means through which youth-talented athletes can progress through the sports system [58]. Nevertheless, it is worth considering that not all swimmers developing in the south have the same level of social support (i.e., family, financial, and affective support; [39]). Thus, not everyone has the opportunity or encouragement to pursue their ambition of becoming a successful senior swimmer.

### 4.1. Practical Implications and Future Directions

In their recent study, Born and colleagues [59] observed that male world-class finalists started achieving higher performance standards from the age of 15–16 compared to their international-class counterparts, who, in contrast, started outperforming national-class swimmers from the age of 13–14. Similarly, Brustio et al. [60,61] found that, in a cohort of European swimmers, most high-performance youth swimmers (i.e., >18 years of age) were not able to retain their status. As such, research has proposed that swimming federations should focus on later talent selection (i.e., after 16 years of age or post-maturation; [62,63]). This would help to distribute resources (i.e., financial, personnel, and infrastructures) among more talented youth athletes [64]. Accordingly, the FIN should carefully reflect on the sociocultural influences and contextual factors embedded into their talent pathway, working to reduce the value-directedness towards competition and hard work from the very first stages, which leads to inequalities in opportunities to develop and represents a form of talent wastage, as reflected in the presence of birth advantages, and focus instead on a more homogenous distribution of resources.

Further investigations of birth advantages within the Italian swimming context should explore swimmers’ careers from a longitudinal perspective to study the existence of performance corridors to obtain a deeper insight into how performance at youth levels, age-group structures, and cultural and contextual environmental factors impact swimmers’ developmental outcomes [48].

Moreover, recent research in youth swimming has shown the efficacy of using corrective adjustment procedures to reduce inequalities caused by RAEs and improve the accuracy of selection decisions [65,66,67,68]. Therefore, in the context of Italian swimming, the use of corrective adjustment procedures and creation of more swimming infrastructures in the south may help combat the proliferation of birth advantages.

### 4.2. Limitations

When interpreting the findings of this study, it is important to take its limitations into account. Firstly, participation in this study was restricted to those who had competed at the U15 or U17 national age-group competition. However, some swimmers could have competed in considerably more races than others and may have achieved more success than others (i.e., medals). Second, this study did not consider the duration of swimmers’ youth career and/or their career trajectories. Third, to analyze the place of early development, this study considered swimmers’ youth clubs at the U15 and U17. A more detailed investigation should have collected swimmers’ first youth clubs to comprehend how contextual features of the environment impact athletes’ very early developmental experiences. Lastly, we only analyzed male swimmers. Since women and girls remain underrepresented throughout sports science research, it is important to study both male and female swimmers, as developmental systems differ; thus, this research may not be applicable in that context.

## 5. Conclusions

This was the first study aiming to investigate how birth advantages (i.e., RAEs and PEDs) affect entry into and progression through the Italian national swimming talent pathway. Part 1 of this study revealed that relatively younger swimmers, developing in north and central Italy, have the greatest probability of competing at the annual U15 national swimming competition. Part 2 of this study explored how birth advantages interact with national-level swimmers’ progression through the model by recording the birthdates and place of early development of Italian youth swimmers who competed at the annual U17 national event and comparing them to the expected distribution based on the U15 cohort. The results revealed more later-born swimmers and a further gap between swimmers developing in north Italy and swimmers developing in south Italy, thus suggesting that progression through the model may be associated with swimmers’ birthdate and place of early development. As such, this study provides a further test of the presence of knock-on effects of relative age, whereby RAEs at successive developmental stages can only be attributed to a residual bias, and presents a new exploration of the underdog hypothesis in the swimming context.

## Data Availability

Data are public and freely accessible at the following link: https://www.federnuoto.it/home/nuoto.html (accessed on 25 June 2024).
